# Improving yield potential in crops under elevated CO_2_: Integrating the photosynthetic and nitrogen utilization efficiencies

**DOI:** 10.3389/fpls.2012.00162

**Published:** 2012-07-19

**Authors:** Surya Kant, Saman Seneweera, Joakim Rodin, Michael Materne, David Burch, Steven J. Rothstein, German Spangenberg

**Affiliations:** ^1^Department of Primary Industries, Biosciences Research Division, Grains Innovation Park, Horsham, VIC, Australia; ^2^Department of Agriculture and Food Systems, The University of Melbourne, Horsham, VIC, Australia; ^3^Department of Primary Industries, Biosciences Research Division, Victorian AgriBiosciences Centre, Bundoora, VIC, Australia; ^4^Department of Molecular and Cellular Biology, College of Biological Science, University of Guelph, Guelph, ON, Canada; ^5^La Trobe University, Bundoora, VIC, Australia

**Keywords:** photosynthesis, nitrogen use efficiency, Rubisco, carbon, nitrogen, elevated CO_2_, yield

## Abstract

Increasing crop productivity to meet burgeoning human food demand is challenging under changing environmental conditions. Since industrial revolution atmospheric CO_2_ levels have linearly increased. Developing crop varieties with increased utilization of CO_2_ for photosynthesis is an urgent requirement to cope with the irreversible rise of atmospheric CO_2_ and achieve higher food production. The primary effects of elevated CO_2_ levels in most crop plants, particularly C_3_ plants, include increased biomass accumulation, although initial stimulation of net photosynthesis rate is only temporal and plants fail to sustain the maximal stimulation, a phenomenon known as photosynthesis acclimation. Despite this acclimation, grain yield is known to marginally increase under elevated CO_2_. The yield potential of C_3_ crops is limited by their capacity to exploit sufficient carbon. The “C fertilization” through elevated CO_2_ levels could potentially be used for substantial yield increase. Rubisco is the rate-limiting enzyme in photosynthesis and its activity is largely affected by atmospheric CO_2_ and nitrogen availability. In addition, maintenance of the C/N ratio is pivotal for various growth and development processes in plants governing yield and seed quality. For maximizing the benefits of elevated CO_2_, raising plant nitrogen pools will be necessary as part of maintaining an optimal C/N balance. In this review, we discuss potential causes for the stagnation in yield increases under elevated CO_2_ levels and explore possibilities to overcome this limitation by improved photosynthetic capacity and enhanced nitrogen use efficiency. Opportunities of engineering nitrogen uptake, assimilatory, and responsive genes are also discussed that could ensure optimal nitrogen allocation toward expanding source and sink tissues. This might avert photosynthetic acclimation partially or completely and drive for improved crop production under elevated CO_2_ levels.

## INTRODUCTION

The human population has just crossed the mark of seven billion, and by the middle of this century it is expected to exceed nine billion (Godfray et al., 2010). To sufficiently feed such a large population, considerable stress will be imposed on increasing crop productivity due to a combination of factors, including shortage of arable land, resource constraints of water and nutrients, changing food habits, use of crop produce for biofuel, and rapid global environmental changes. Although, agronomic and breeding efforts in the past five decades have achieved a linear increase in food productivity, a further ability to increase or even sustain the crop yield and quality is uncertain in the face of rapid global environmental change (Rothstein, 2007; Tester and Langridge, 2010). The environmental changes are coincident with increasing biotic and abiotic threats such as heat and water stress, newer insect-pests as well as diseases and rising greenhouse gases including elevated CO_2_. Among these, atmospheric CO_2_ levels are increasing linearly over time; present atmospheric CO_2_ has increased from 280 to 390 μmol mol^-1^ since 1800, and is expected to double by the end of the twenty-first century (IPCC, 2007). Plants could adapt to these elevated levels through photosynthetic conversion of high CO_2_ into increased growth and productivity. However, the potential for different plant species to assimilate higher CO_2_ concentrations and their consequences is not yet fully understood.

**FIGURE 1:**
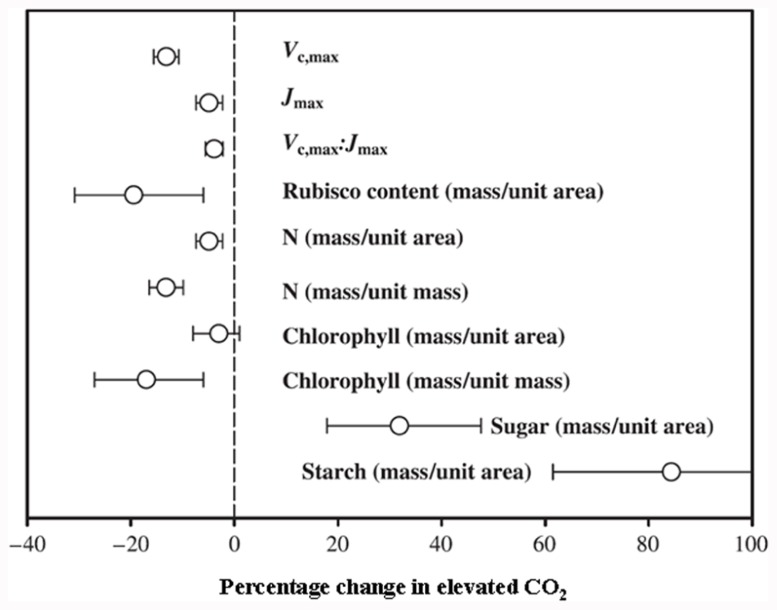
**Mean response of maximum carboxylation rate (*V*_c,max_), maximum rate of electron transport (*J*_max_), ratio of *V*_c,max_:*J*_max_, and Rubisco, N, chlorophyll, sugar and starch contents**. Rubisco, sugar and starch contents reported on area basis. N and chlorophyll contents reported on both area and mass basis at 95% confidence level. Number of species, FACE experiments and individual observations for each response are given in Ainsworth and Long (2005). LAI, leaf area index; DMP, above ground dry matter production (Reproduced with permission).

Carbon (C) and nitrogen (N) are the key structural elements for plant growth and constitute ~45 and ~5% of plant dry matter, respectively (Ho, 1976; Marschner, 1995). The maintenance of optimum C and N balance (often referred as the C/N ratio) within plants as well as externally in soil or growth media is essential for optimal plant growth and development (Paul and Driscoll, 1997; Martin et al., 2002; Malamy, 2005; Wingler et al., 2006; Zhang et al., 2007). Simultaneous improvement of both C and N utilization efficiencies is of utmost importance, given the rising atmospheric CO_2_ levels and the necessity to lower input costs and reduce environmental pollution due to excessive use of nitrogenous fertilizers. Therefore, fine-tuning of genetic changes leading to metabolic adjustments of both C and N will be required in order to effectively harness excess C from elevated atmospheric CO_2_ and to simultaneously maintain an optimal C/N balance. The primary effects of elevated CO_2_ levels in most crop plants (especially C_3_ plants) include increased plant biomass accumulation, although initial stimulation of net photosynthesis rates for most C_3_ plants is only temporal, and they fail to sustain the maximum stimulation (though higher than ambient CO_2_ level) over longer exposure periods (months to years). This phenomenon is called CO_2_ or photosynthesis acclimation (Long et al., 2004; Reich et al., 2006; Bloom et al., 2010). This photosynthesis acclimation (initial stimulation followed by a partial reversal or stabilization at a lower rate) under elevated CO_2_ is accompanied by a decrease in carboxylation of ribulose-1,5-bisphosphate carboxylase/oxygenase (Rubisco), a decrease in N concentration, a reduced stomatal conductance, and an increase of starch accumulation (**Figure 1**; Nakano et al., 1997; Geiger et al., 1999; Stitt and Krapp, 1999; Bloom et al., 2002, 2010; Long et al., 2004; Ainsworth and Long, 2005; Leakey et al., 2009).

In C_3_ plants, Rubisco is the key chloroplast enzyme (comprising ~50% of total cellular proteins) involved in photosynthesis through catalyzing the carboxylation of ribulose-1,5-bisphosphate (RuBP) during capture and fixation of atmospheric CO_2_. This CO_2_ is later converted into sugars, the major building blocks for plants. In addition to the involvement of Rubisco in C metabolism, it is also a major storage protein for N (Makino and Osmond, 1991; Mae et al., 1993). This stored N is further utilized by the plants’ reproductive components when Rubisco degradation is initiated during leaf senescence. The consequences of elevated CO_2_ on plants are 2-fold; the decreased Rubisco level becomes a rate-limiting factor for photosynthetic efficiency compounded by a reduction in the available N pool. Genetically engineered plants producing increased levels of Rubisco protein could potentially improve CO_2_ fixation. However, plants under these conditions would require additional N for increased Rubisco production. Additionally, maintenance of an optimum C/N ratio within the plant is essential for efficient metabolism of C and N, optimal growth, and sustained quantitative and qualitative yield. High C status (specifically carbohydrates) due to increased CO_2_ levels would increase the C/N ratio with lower N levels resulting in lower protein content, thus reducing grain quality particularly in cereal crops.

There is mounting evidence that the yield potential of many crops is limited by their capacity to exploit sufficient C during their lifecycle, limiting grain size and quantity (Fischer et al., 1998). “C fertilization” through increased CO_2_ levels would be ideal for yield increase; however, photosynthetic acclimation restricts the plants’ ability to exploit elevated atmospheric CO_2_. In this review, we discuss the underlying causes of this stagnation in yield progress and explore the possibilities of improving the photosynthetic machinery in plants, combined with enhanced nitrogen use efficiency (NUE) under elevated CO_2_ conditions. Engineering N uptake, assimilatory and responsive genes would ensure optimal N allocation toward expanding source and sink tissues under elevated CO_2_ levels as well as improving grain yield and quality.

## C_3_ AND C_4_ PHOTOSYNTHETIC MECHANISMS

Photosynthesis is the process whereby light is harvested by the chloroplast thylakoids of the leaf and other photosynthetic structures. The resultant chemical energy (ATP and NADPH) is used to fix atmospheric CO_2_, either directly via Rubisco (C_3_ photosynthesis), or indirectly after primary fixation by phosphoenolpyruvate carboxylase (PEPC). C fixed through this mechanism is subsequently re-released into adjacent cells which are not in direct communication with atmospheric CO_2_ (C_4_ photosynthesis). The majority of crop species (rice, wheat, grain legumes, canola, and all root crops) and ~85% of terrestrial plants use C_3_ photosynthesis, while C_4_ crops are a minority, represented predominantly by maize, sorghum, and sugarcane among economically important crops (Ehleringer et al., 1991).

The Rubisco enzyme, which is fundamental to C fixation in both C_3_ and C_4_ plants, displays a high affinity to O_2_, and its inability to distinguish it from the CO_2_ molecule results in unnecessary O_2_ uptake, especially under hot and arid conditions. This oxygenation activity produces phosphoglycolate molecules, which are then broken down in a process referred to as photorespiration, an energy-consuming and wasteful process (Kajala et al., 2011). Photorespiration has been identified as the bottleneck preventing C_3_ plants from achieving full photosynthetic potential due to competition between CO_2_ and O_2_ at the C fixation site on the Rubisco enzyme. Whereas, C_4_ photosynthesis evolved to ameliorate photorespiration by utilizing two distinct cell types not involved in the C_3_ photosynthesis: mesophyll cells (MC) and bundle sheath cells (BSC), which are rarely more than one cell distant from each other, allowing ease of molecular transport between the two. The tissues within these cells are arranged concentrically relative to the surrounding vascular tissue, a structure characteristic of C_4_ plants known as Kranz anatomy (Muhaidat et al., 2007; Sage et al., 2012). When atmospheric CO_2_ is assimilated into the MC, carbonic anhydrase and PEPC hydrate and fix C molecules as oxaloacetate. This reaction has no affinity to O_2_ and is highly efficient (Sheen, 1999). The resulting C_4_ acid is decarboxylated within the BSC, delivering higher concentrations of CO_2_ directly to the Rubisco enzyme while minimizing the oxygenation of Rubisco. The increased concentration of CO_2_ at the site of Rubisco activity maximizes photosynthetic efficiency. These evolutionary adaptations in C_4_ plants provide an advantage over C_3_ photosynthesis while potentially improving water and nutrient use (Kajala et al., 2011). Zhu et al. (2008) reported a 60% increase in maximum photosynthetic efficiency in C_4_ plants compared to C_3_ plants. C_4_ plants can photosynthesize with ~50% greater water use efficiency, as C_4_ photosynthesis can assimilate an equivalent amount of CO_2_ with only half the stomatal conductance (Sage and Kubien, 2003; von Caemmerer and Furbank, 2003). Under N-limiting conditions, C_4_ plants also out-compete C_3_ plants, as they require less Rubisco to harness a similar amount of C due to increased photosynthetic efficiency (Sage and Kubien, 2003).

**FIGURE 2:**
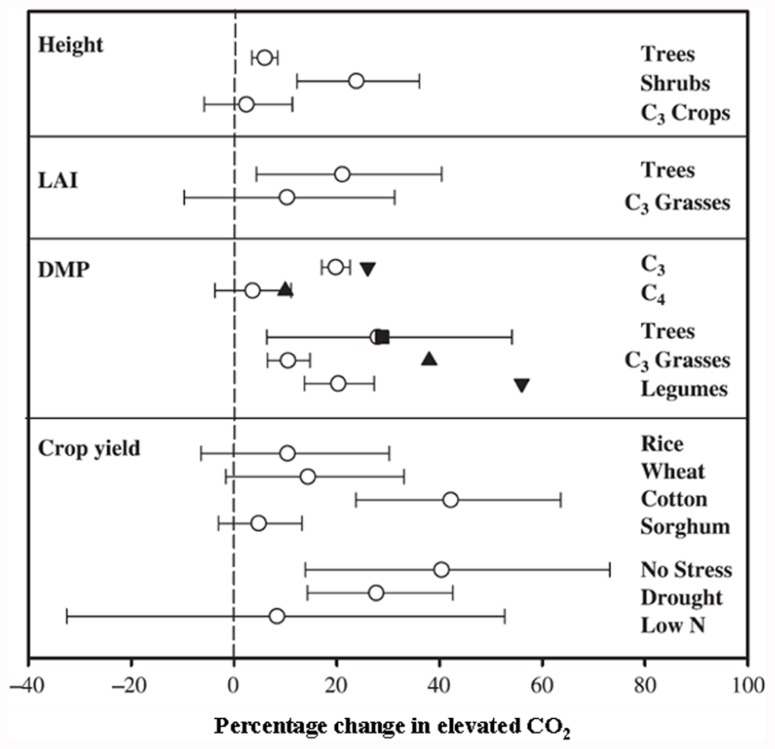
**Responses to elevated CO_2_ of different plant species and experimental conditions on growth and yield variables**. Results from: **◯**, a meta-analysis of various species (Ainsworth and Long, 2005); ∎, a meta-analysis of tree species (Curtis and Wang, 1998); ▲, a meta-analysis of C_4_ plants and C_3_ grasses (Wand et al., 1999). ▼, a meta-analysis of C_3_ and legumes (Jablonski et al., 2002). Number of species, FACE experiments and individual observations for each response are given in Ainsworth and Long (2005; reproduced with permission).

Since C_4_ plants are photosynthetically saturated at current CO_2_ conditions, predicted rises in atmospheric CO_2_ would have no major impact on their C fixation rate, biomass production, and yield (**Figure 2**; Cure and Acock, 1986; Ainsworth and Long, 2005). In contrast, as C_3_ plants are not photosynthetically saturated at present CO_2_ levels, photosynthesis, biomass, and subsequent yields should increase with elevated atmospheric CO_2_. The understanding of the biochemical and molecular nature of C_3_ and C_4_ photosynthesis provides a valuable tool for crop improvement in the twenty-first century, particularly with respect to improving C assimilation in C_3_ plants and reducing the impact of photosynthetic acclimation.

## IMPACTS OF ELEVATED CO_2_

### LEAF PHOTOSYNTHESIS, GROWTH, AND YIELD

The present atmospheric CO_2_ concentration of 390 μL CO_2_ L^–1^ limits the rate of photosynthesis in C_3_ plants (Farquhar et al., 1980; Farquhar and Sharkey, 1982), and presumably lower concentrations of CO_2_ in the recent past were even more limiting. Laboratory and field studies have shown that photosynthetic rates of C_3_ plants were approximately doubled when plants grown at about 380 μL CO_2_ L^–1^ were exposed to 700 μL CO_2_ L^–1^ (Ainsworth and Long, 2005). This increase in photosynthetic rate as atmospheric CO_2_ rises is primarily due to increase in Rubisco carboxylation capacity. Rubisco has an affinity for O_2_ as well as CO_2_ (Badger and Price, 2003); consequently, at 21% O_2_ and 390 μL CO_2_ L^–1^, a considerable amount of energy is wasted in the photorespiratory carbon oxidation cycle (PCO). This reduces photosynthetic rates by about 40% from the optimum level (Sharkey, 1985). Increasing the ambient CO_2_ concentration increases the ratio of CO_2_ to O_2_ at the site of fixation in the chloroplast, favoring PCR over PCO, and thus photosynthetic rates are increased in C_3_ plants. The limitation of photosynthesis imposed by Rubisco is referred to as the limitation due to supply and utilization of CO_2_ (Farquhar and Sharkey, 1982). Two other limitations were also identified: the supply and utilization of light and the utilization of triose phosphate. The former can be caused by low photon flux densities or inability to convert light energy to chemical energy. Triose phosphate is the end product of photosynthesis and can be formed into starches and sugars or utilized as a direct source of chemical energy. Limitation occurs when there are insufficient sinks for sucrose (Stitt and Schulze, 1994), thus reducing conversion to sugar and inhibiting photosynthesis. These three limitations to leaf photosynthesis were first identified in plants that were grown at a given CO_2_ concentration and then transferred to different CO_2_ concentration during measurement of photosynthesis (Drake et al., 1997). However, when plants are exposed to high CO_2_ for extended period, the photosynthetic rates slow down due to the so-called “acclimation” response (Long et al., 2004; Reich et al., 2006). This is thought to result from direct effects of sucrose on the transcription of genes encoding proteins involved in CO_2_ fixation and electron transport activity (Moore et al., 1999).

The effect of elevated CO_2_ on plant growth and yield has been studied in both controlled and field conditions, with the latter referred to as the Free Air Carbon dioxide Enrichment (FACE) system. The controlled conditions might produce larger artifacts, whereas FACE produces an environment similar to field conditions. The differential plant response under the two conditions has been reported. For example, Ainsworth et al. (2008) suggested a 14% yield increase in FACE and a 31% increase in controlled conditions in different plant species when CO_2_ was raised from ~373 to ~570 μmol moL^–1^. Generally, elevated CO_2_ increases photosynthesis, resulting in increased dry matter accumulation, leaf area, and plant height in trees and shrubs and to some extent in C_3_ plants (**Figure 2**; Ainsworth and Long, 2005). The yield increase in C_3_ crops under elevated CO_2_ is variable and dependent on other environmental factors such as water, temperature, and soil N (Ainsworth and Long, 2005). Irrespective of photosynthetic machinery, a yield increase requires a concomitant increase in sink capacity to match the source activity. The initial response of C_3_ plants to elevated CO_2_ is an increase in photosynthetic rate; however, due to the acclimation phenomenon this stimulation is not always maintained at the maximal level when plants are exposed to elevated CO_2_ for a longer period. This partial reversal of photosynthesis and settling at lower than maximal level could be ascribed to (i) reduced stomatal conductance resulting in depletion of intercellular CO_2_, leading to reduced CO_2_ supply to the photosynthetic machinery, and (ii) reduced rates of electron transport to Rubisco carboxylation (**Figure 1** and also discussed in the following sub-section). Lower activation state and reduced concentration of Rubisco leads to changes in C assimilation and alters the whole plant N metabolism. Thereby, biochemical adjustments occur from the cellular to whole plant level in response to elevated CO_2_, accompanied by growth, development, and yield changes.

### MOLECULAR CHANGES IN PLANTS

Rubisco is the rate-limiting enzyme in photosynthesis and its synthesis and degradation is affected by environmental factors such as temperature, light intensity, soil N, and atmospheric CO_2_. Prolonged exposure to elevated CO_2_ results in reduced Rubisco content and Rubisco activity (Moore et al., 1999; Aranjuelo et al., 2011; Seneweera et al., 2011). A concomitant reduction in the transcript level of genes encoding proteins involved in photosynthesis, including *small subunit of Rubisco* (*RbcS*), *large subunit of Rubisco* (*RbcL*), and *Rubisco activase* (*Rca*), has been observed in different plants (Nie et al., 1995; Cheng et al., 1998; Moore et al., 1998, 1999; Stitt and Krapp, 1999). In contrast, in expanding rice leaf blades there was no significant difference in *RbcS* transcript level between ambient and elevated CO_2_ levels (Aoki et al., 2003). This can be correlated with changes in Rubisco concentration during leaf development, with a rapid increase in Rubisco protein during leaf expansion, reaching a maximum when the leaf is fully expanded and a gradual decline with the onset of leaf senescence (Seneweera and Conroy, 2005; Imai et al., 2008). The decline in Rubisco and subsequent photosynthesis acclimation in plants under elevated CO_2_ could be attributed to two processes. It could be due to carbohydrate sink limitation since plants grown under CO_2_ enrichment initially assimilate more CO_2_ than they can incorporate in their sink tissues, and as a feedback response plants diminish CO_2_ assimilation by reducing levels of Rubisco and other proteins (Long et al., 2004). Previous reviews have reported that feedback repression of the *RbcS* and *RbcL* genes by soluble carbohydrates accumulation leads to a decline in Rubisco protein levels (Moore et al., 1999). Alternatively the C/N ratio usually increases under elevated CO_2_ (Geiger et al., 1999; Luo et al., 2004; Taub and Wang, 2008; Bloom et al., 2010), since N is a key constituent of Rubisco it becomes a rate-limiting factor for Rubisco synthesis (Nakano et al., 1997; Seneweera et al., 2011).

### NUTRITIONAL CHANGES IN PLANTS

Elevated CO_2_ stimulates higher photosynthesis and an increased growth rate, which is required to match with an increased demand for nutrients. This may vary between plant species, nutrient availability, and the nutrient element in question. Among different nutrient elements, maintaining the C/N balance is important for optimal plant growth. For instance, under a higher C/N ratio in soil or growth media, there is a reduced uptake of N in plants, leading to reduced grain quality in cereals due to lower grain protein content. In cereals such as wheat, rice, and barley, a decrease of up to 15% grain protein was observed under elevated CO_2_, with an overall decrease in amino acid concentrations (Taub et al., 2008; Wieser et al., 2008; Högy et al., 2009). A decrease in cereal grain quality and a reduced protein composition may have serious health and economic implications. The spatial leaf N content has a strong correlation with Rubisco content in rice leaves, suggesting their inter-dependency with net photosynthetic rates (Seneweera, 2011). Leaf N allocation clearly declines under elevated CO_2_, accompanied by lower chlorophyll content, as both are closely linked (**Figure 1**; Conroy and Hocking, 1993; Nakano et al., 1997; Ainsworth and Long, 2005; Leakey et al., 2009), also discussed in a later section. Other macro- and micro-nutrient concentrations change under elevated CO_2_ conditions, though with lesser implications compared to N (Högy et al., 2009; Erbs et al., 2010). Potassium and phosphorous contents can increase or decrease depending upon growth conditions. Significantly lower levels of sodium, calcium, magnesium, sulfur, iron, zinc, manganese, and aluminum contents have been observed in wheat grain under elevated compared to ambient CO_2_ (Högy and Fangmeier, 2008).

## STRATEGIES FOR IMPROVING PHOTOSYNTHETIC RATES IN C_3_ PLANTS

Increase in net photosynthesis per unit leaf area is important for increasing crop production to meet the world food demand. To improve photosynthesis rates in C_3_ plants several approaches have been used, for example introducing C_4_ like characteristics into C_3_ cells (Kajala et al., 2011; Miyao et al., 2011; Peterhansel, 2011); introducing a CO_2_/HCO_3_ pump protein into chloroplast membranes from cyanobacteria (Price et al., 2008); introducing new catabolic pathways into plastids that bypass the photorespiratory recycling Rubisco oxygenation product, 2-phosphoglycolate, and concomitantly releasing CO_2_ into the stroma (Kebeish et al., 2007); and also improving the Rubisco kinetic characteristics. Some opportunities to improve photosynthetic efficiency in C_3_ plants are discussed here.

C_4_ photosynthesis has been identified to be evolving independently at least 66 times in 19 different families of angiosperms (Sage et al., 2011) with 21 of these lineages displaying the intermediate C_3_–C_4_ photosynthetic characteristics (Brown and Hattersley, 1989; Edwards et al., 2004). The evolution of C_4_ from C_3_ photosynthesis involves a number of intermediate steps, while the enzymes and structures present in C_4_ plants are also present in C_3_ plants in some form (Ehleringer et al., 1991). This is advantageous for plant biologists attempting to engineer C_4_ pathways in C_3_ plants, through the identification of genotypes expressing some degree of cellular similarities to C_4_ plants, such as high numbers of chloroplasts in the BSC, and separation of MC by only a single cell (Furbank et al., 2009). Partial C_4_ cycles genes have been introduced into rice, potato, and tobacco (Kajala et al., 2011) without incorporating the complete Kranz anatomy, which would require targeting multiple genes. The Kranz anatomy of C_4_ plants is generally considered to be too complex to engineer into C_3_ cells. However, two species of the family Chenopodiaceae were found to have a C_4_ photosynthesis system contained within a single chlorenchyma cell in the absence of Kranz anatomy. The cells performed the same role as Kranz anatomy by partitioning themselves into two cytoplasmic compartments (Edwards et al., 2004). Each of these cells performs a function analogous to the MC and BSC in Kranz anatomy, serving to concentrate C around Rubisco. This has provided hope that C_4_ like photosynthesis can be introduced into C_3_ plants in the absence of full Kranz anatomy. Specific genes have been suggested by Miyao (2003) in C_3_ plants very similar to those in C_4_; however their expression level is very low are thought to serve housekeeping functions (Kajala et al., 2011). Overexpression of native C_3_ genes or homologous C_4_ genes could possibly be useful for improving photosynthetic efficiency in C_3_ plants. Attempts have been made to engineer single-celled and two-celled photosynthetic pathways with expression of specific genes into rice (Kajala et al., 2011; Miyao et al., 2011). Four C_4_ genes, *PEPC*, malate dehydrogenase (*MDH*), NADP-malic enzyme (*NADP-ME*), and NADP-malate dehydrogenase (*NADP-MDH*) have been engineered in rice (Taniguchi et al., 2008; Miyao et al., 2011). However, a functional C_4_ cycle in leaves of C_3_ species has not yet achieved. In addition, the negative effects of engineering C_4_ genes in C_3_ plants have been reported, such as overexpression of maize NADP-ME in rice that led to enhanced photoinhibition of photosynthesis and pleiotropic effects (Tsuchida et al., 2001), stunted transgenic plants due to generation of futile cycles and improper circadian regulation of genes (Taniguchi et al., 2008; Kajala et al., 2011). To achieve C_4_ photosynthetic pathway operational in C_3_ plants is an enormous challenge and would require: careful selection and engineering of multiple genes encoding for both C_4_ photosynthetic genes and transporters of C_4_ metabolites, driving the optimal expression of genes, site specific expression of selected genes such as in MC or BSC, choice of coding sequence since C_4_ genes have acquired changes in coding regions during evolutionary process, and proper regulation of C_4_ enzymes (Kajala et al., 2011; Miyao et al., 2011; Peterhansel, 2011).

Cyanobacteria are a phylum of bacteria that obtain energy through photosynthesis and are the ancestors of chloroplasts in eukaryotic cells (Raven and Allen, 2003). Cyanobacteria are highly efficient for producing biomass from inorganic C (Nogales et al., 2012). As a photosynthetic organism, cyanobacteria have evolved a very efficient mechanism for converting CO_2_ into HCO_3_^–^, an important step in transporting C into the chloroplast stroma. The carbon concentrating mechanism (CCM) of cyanobacteria delivers several-fold higher CO_2_ to Rubisco sites compared to C_3_ photosynthesis. There have been a number of attempts made to introduce CCM into C_3_ plants, but so far limited progress has been made (Hibberd et al., 2008; Peterhansel et al., 2008). A cyanobacterial gene *ictB* has been linked to HCO_3_^–^ accumulation within cyanobacteria, and when expressed in transgenic *Arabidopsis* and tobacco resulted in a significant increase in photosynthetic rates (Lieman-Hurwitz et al., 2003). The expression of such genes in crop plants could lead to significant yield increases by developing a C_4_ style C concentration mechanism within plants currently exhibiting C_3_ anatomy. Only a small subset of genes would need to be transferred to C_3_ crop species, and specialized anatomy and morphology may not be required; engineering such changes is within the scope of the current genomic technology.

## INCREASING THE N AVAILABILITY IN PLANTS TO IMPROVE YIELD AND QUALITY UNDER HIGHER “C FERTILIZATION” THROUGH ELEVATED ATMOSPHERIC CO_2_

As macronutrients, both C and N have pivotal roles in plant growth. Additionally, these nutrients can act as signaling molecules influencing several cellular processes through regulation of gene expression in plants. It has been suggested that about half of the *Arabidopsis* genome is regulated by C, N, or C/N interaction (Palenchar et al., 2004; Gutierrez et al., 2007). An optimum C/N ratio is required for smooth operation of several growth, developmental, and biochemical processes such as seedling development, root architecture, lateral root development, flowering time, senescence progression, photosynthesis, and regulation of C and N assimilation (Paul and Driscoll, 1997; Corbesier et al., 1998; Martin et al., 2002; Malamy, 2005; Wingler et al., 2006; Gutierrez et al., 2007; Zhang et al., 2007).

Photosynthetic acclimation and a decrease in Rubisco levels under elevated CO_2_ are pre-dominant in N-limited plants compared to sufficient N-supplied plants (Miglietta et al., 1996; Riviere-Rolland et al., 1996; Rogers et al., 1996; Geiger et al., 1999; Stitt and Krapp, 1999; Ainsworth et al., 2003; Reich et al., 2006). Photosynthetic acclimation is also associated with plants acquiring and assimilating insufficient N at elevated CO_2_, leading to N limitation in plant tissues and subsequently lower C acquisition. Under low N these response are much greater at elevated CO_2_ (Geiger et al., 1999; Luo et al., 2004; Taub and Wang, 2008; Bloom et al., 2010). Also, the exogenous supply of C as sucrose to N-limiting plants led to accumulation of higher carbohydrate and a decrease of Rubisco and chlorophyll content (Paul and Stitt, 1993; Paul and Driscoll, 1997). The decrease of N content in plant biomass at elevated CO_2_ compared to ambient CO_2_ is usually within the range of 10–15% (**Figure 1**; Jablonski et al., 2002; Kim et al., 2003; Ainsworth and Long, 2005; Seneweera, 2011; Seneweera et al., 2011). This decrease could be due to a range of factors. First, it could involve a dilution of N in plant tissues by an increased flux of photosynthate compounds from excess carbohydrate accumulation. Second, there could be a decreased transpirational driven flow of N due to reduced stomatal conductance. Third, there could be decreased N uptake due to both source effects (soil-root specific) and reduced demand (down-regulation of photosynthetic enzymes). Fourth, a decreased N assimilation capacity could be involved. Finally, there could be a reduced electron flow for nitrate assimilation. In particular, the photosynthetic C reduction cycle and nitrate assimilation compete for electrons from photosynthesis, and since CO_2_ assimilation is favored, this results in a reduced N influx (Bloom et al., 2002, 2010; McDonald et al., 2002; Luo et al., 2004; Taub and Wang, 2008).

The decrease in N content in plants grown under elevated CO_2_ aggravates N-limiting conditions in the following years, as plant leaves senescence and drops to the soil, thus increasing microbial immobilization of N. Due to a high C/N ratio, the availability of N to plants further declines as more N is fixed in soil microbes. Consequently, this leads to progressive N limitation in most agricultural cropping systems (Ball, 1997; Luo et al., 2004). This suggests that N supply needs to be matched with higher C assimilation under elevated CO_2_, requiring new N management strategies in agriculture. Legumes have the potential to respond maximally to higher CO_2_ due to their N-fixing capacity matching the excess C gain at elevated CO_2_. This allows maximum utilization of sink capacity (Rogers et al., 2006). This improved N assimilation has increased photosynthetic stimulation and higher productivity in legumes compared to non-legumes under elevated CO_2_ (Zanetti et al., 1996; Ainsworth et al., 2002; Rogers et al., 2006). Introducing N-fixation capabilities in non-leguminous plants has always been an attractive prospect, which could close the N acquisition gap, allowing efficient CO_2_ capture and maximizing yield gains under elevated atmospheric CO_2_. Otherwise, in non-leguminous plants additional N fertilization would be required to obtain similar yield benefits, which might not be economically and environmentally feasible. Maximization of plant growth under elevated CO_2_ conditions without additional N application would result in yield increases at no additional cost of fertilizer or risk to the environment. There is a large opportunity to efficiently utilize the existing N supply in most agricultural soils given that most cereal crops have a NUE of less than 40%. Therefore, over 60% of soil N is lost through a combination of leaching, surface runoff, de-nitrification, volatilization, and microbial consumption. It is estimated that a 1% increase in NUE could save ~$1.1 billion annually. Hence, developing crop varieties with a higher NUE would minimize the loss of N, reduce environmental pollution and decrease input cost (Kant et al., 2011). NUE has been defined in several ways (Good et al., 2004); the most simple and practical is yield per unit of N available in the soil. Coordinated efforts are required to increase N uptake, assimilation, and/or remobilization efficiency for improved NUE. During vegetative growth, N uptake is dedicated to storage and assimilation into amino acids where developing leaves and shoots act as sinks for N. During reproductive development, N assimilation and remobilization becomes more prominent, and leaves and shoots act as an N source, supplying amino acids for reproductive organs.

Attempts have been made to manipulate the expression of different nitrate and ammonium transporters and assimilatory genes, mostly in model plants and some crop plants (Good et al., 2004; Kant et al., 2011). Higher or lower N contents led by overexpression or mutation of genes for N transporters have been reported in several studies in *Arabidopsis* (Geelen et al., 2000; De Angeli et al., 2006; Chopin et al., 2007). However, evidence of improved yield or NUE in crop plants attributed to modification of N responsive genes is limited, with little improvement in some traits. For example, transgenic wheat plants displayed enhanced N assimilation capacity by overexpression of *Glutamine Synthetase* (*GS1*; Habash et al., 2001). Overexpression of the *GS1.3* gene in maize resulted in an increase of 30% kernel number (Martin et al., 2006). The limited benefits of modifications of such genes could be ascribed to (i) lack of suitable combinations of promoters and regulatory elements for specific expression of the target genes that can match with a certain growth stage, plant tissue type, or environmental conditions, and (ii) limited sink capacity in crop plants to assimilate and utilize additional N for increased growth and yield. Additional C skeleton and C assimilation would be required to expand sink capacity where surplus N can be incorporated. Overexpression of genes for N uptake, transport, and assimilation would ensure increased availability of N content and amino acids utilized for photosynthetic machinery which mainly control plant growth and development. Simultaneously, elevated atmospheric CO_2_ would ensure adequate C supply for enhanced plant growth. Hence, orchestrating coordinated efforts for growing C_3_ plants with increased NUE and better C assimilation capacity under elevated CO_2_ would be an effective strategy for avoiding CO_2_ or photosynthesis acclimation, leading to higher growth rates, yield, and quality in cereals.

## CONCLUDING REMARKS

Plant growth is typically stimulated at elevated CO_2_, but often decreases with time, due to relaxation of photosynthesis to a lower rate under exposure to elevated CO_2_ over longer periods. The sustained and maximal stimulation of growth at elevated CO_2_ requires acquisition of additional N to maximize increased C assimilation. Coordinated efforts for increasing photosynthetic efficiency, enhancing sink capacity, and improving N uptake would potentially increase grain yield under rising atmospheric CO_2_. A marginal increase in crop growth and yield has been reported in several FACE experiments. Nevertheless, improving NUE and N uptake in crop plants could partially avert the limitations of both photosynthetic acclimation and reduced grain quality under elevated CO_2_ levels. The major private plant biotechnology companies are attempting to develop improved NUE transgenic lines in their research and development strategies. This would be an effective method and would reinforce their strategy for improved grain quality under commonly accepted climate change scenarios. In the past 15 years (1996–2010), the accumulated global land area for transgenic crops exceeded one billion hectares grown by over 15 million farmers (James, 2010). The use of genetically engineered crops has increased farmer profit, reduced herbicide and pesticide usage, and reduced chemical impact on the environment, which has mainly been achieved through single gene modifications. However, the traits of improving NUE and enhanced crop response to elevated CO_2_ are more complex and would require stacking of multiple modified genes. Efficient management of ammonium and nitrate application could also facilitate benefits of increased yield and sustained grain quality under forecasted atmospheric CO_2_ elevation. However, careful manipulations of N-responsive genes provide the greatest global advantages, since additional nitrogenous fertilizers pose undesirable economic and environmental threats.

## Conflict of Interest Statement

The authors declare that the research was conducted in the absence of any commercial or financial relationships that could be construed as a potential conflict of interest.
